# A Smart Tongue Depressor-Based Biosensor for Glucose

**DOI:** 10.3390/s19183864

**Published:** 2019-09-11

**Authors:** Xiaojin Luo, Weihua Shi, Yiqun Liu, Pengju Sha, Yanan Chu, Yue Cui

**Affiliations:** College of Engineering, Peking University, Beijing 100871, China

**Keywords:** tongue depressor, biosensor, salivary glucose, carbon nanotube, enzyme, hydrogen peroxide

## Abstract

The development of new bioelectronic platforms for direct interactions with oral fluid could open up significant opportunities for healthcare monitoring. A tongue depressor is a widely used medical tool that is inserted into the mouth, where it comes into close contact with saliva. Glucose is a typical salivary biomarker. Herein, we report—for the first time—a tongue depressor-based biosensor for the detection of glucose in both phosphate buffer and real human saliva. Carbon nanotubes (CNTs) are attractive electronic materials, with excellent electrochemical properties. The sensor is constructed by printing CNTs and silver/silver chloride (Ag/AgCl) to form three electrodes in an electrochemical cell: Working, reference, and counter electrodes. The enzyme glucose oxidase (GOD) is immobilized on the working electrode. The glucose detection performance of the sensor is excellent, with a detection range of 7.3 μM to 6 mM. The glucose detection time is about 3 min. The discretion between healthy people’s and simulated diabetic patients’ salivary samples is clear and easy to tell. We anticipate that the biosensor could open up new opportunities for the monitoring of salivary biomarkers and advance healthcare applications.

## 1. Introduction

The development of new types of biosensors has attracted considerable interest in various healthcare, environmental monitoring, and defense applications [1,2,3]. Saliva is easy to obtain using noninvasive methods, and it contains a variety of biomarkers related to diseases or health statuses [4,5,6,7]. Thus, the development of a simple method for the detection of salivary biomarkers is highly desirable. Glucose is a typical salivary compound, and it can be used as a typical analyte for the construction of biosensors [8,9,10].

Many biosensors on conventional substrates, such as ceramics and glass, have been developed [11,12,13]. Recently, the potential utility of several types of nonconventional substrates for the detection of glucose have been studied. These include cotton fabric [14], plastic film [15], plastic centrifuge tubes [14], and contact lenses [16]. A variety of electronic materials have also been studied for the construction of sensing electrodes. These materials include carbon graphite [14,17,18], carbon nanotubes (CNTs) [19,20], graphene [21], polymers [22,23], and indium tin oxide (ITO) [24]. Many of these materials have shown excellent biosensing performances. For example, studies have shown that CNTs, a typical type of electronic material, have excellent electrochemical properties and that they can be used for the construction of amperometric biosensors [25,26].

A tongue depressor is a widely used medical tool that is inserted into the mouth, where it comes into close contact with saliva [27,28]. It is cost-effective, lightweight, and convenient to use for frequent examinations [29]. A tongue depressor could provide a unique platform for building biosensing devices. However, to the best of our knowledge, there are no reports on the construction of any medical-related devices on a tongue depressor.

In this work, we present—for the first time—a tongue depressor-based amperometric biosensor for the determination of glucose. Since a tongue depressor is a commonly used oral medical tool, it can directly contact and detect human saliva. For example, for diabetic patients, the glucose concentration can be tested by placing the tongue depressor-sensor in the mouth instead of using other invasive methods. The sensor is constructed based on printing CNTs and Ag/AgCl to form three electrodes in an electrochemical cell: A CNT working electrode, an Ag/AgCl reference electrode, and a CNT counter electrode. Glucose oxidase (GOD) is immobilized on the working electrode. The reference electrode provides a stable potential. The working electrode and the counter electrode form a circuit. A constant potential is applied between the working electrode versus the reference electrode, and the current in the circuit is measured. The oxidation of glucose is catalyzed by GOD to produce H_2_O_2_. H_2_O_2_ generates a current response, as the detectable signal, via the oxidation reaction at the working electrode. A calibration curve is plotted between the current reponse and the concentration of glucose. We also tested real human saliva with and without added glucose to prove our sensor’s capability in distinguishing between healthy people and those who suffer from diabetes.

## 2. Materials and Methods

### 2.1. Apparatus and Chemicals

A potentiostat CHI 660 was purchased from CH Instrumentation, Inc. (Shanghai, China). A UV ozone cleaner was obtained from Ossila Limited (Sheffield, UK). Ag/AgCl ink with a 50:50 mass ratio was purchased from Gwent Electronic Materials Ltd. (Pontypool, UK). Carbon nanotubes (optical density: 10–20 nm, length: 10–30 μm, ash: <1.5 wt%, specific surface area: >200 m^2^/g, electrical conductivity: >100 s/cm, purity: >95%) were purchased from Xfnano (Nanjing, China). Toluene was purchased from Beijing Tongguang Fine Chemical Company (Beijing, China). Glucose oxidase was obtained from Toyobo Co., Ltd. (Osaka, Japan). Glucose anhydrous was purchased from Tianjin Zhiyuan Reagent Company, Ltd. (Tianjin, China). Glutaraldehyde and hydrogen peroxide were purchased from Sigma-Aldrich Inc. (Beijing, China). Heating glue was acquired from Xnyhc, Inc. (Beijing, China). Artifical saliva was purchased from Yuanye Biotechnology Co. (Shanghai, China).

### 2.2. Sensing Electrode Preparation

First, three parallel rectangles were drawn on the tongue depressor to mark the positions of three electrodes, each with a length of 20 mm, a width of 2 mm, and 2 mm apart from each other. The tongue depressor was then covered with a thin layer of epoxy and dried at room temperature (27 °C) for 24 h to ensure that it was totally waterproof. The working and counter electrodes were prepared manually with CNT (0.1 mg/mL in toluene; CNT: electrical conductivity >100 s/cm, optical density: 10–20 nm, length: 10–30 μm, ash: <1.5 wt%, specific surface area: >200 m^2^/g, purity: >95%). The reference electrode was prepared with Ag/AgCl ink (50:50, Gwent Electronics Materials Ltd.). Scotch tapes were used to cover the area between and around the three rectangles, leaving out the space for the electrodes. The CNT solution was carefully dropped on the locations for the working and counter electrodes with an autopipette. Then, the Ag/AgCl ink was painted on the location for the reference electrode with a foam-tipped swab. The printed electrodes were dried in an oven at 80 °C for half an hour, and then cooled at room temperature. A chamber was casted with a glue gun at the front end of the electrodes where solution would be added. The device was further treated with a UV ozone cleaner for 20 min to change the hydrophobic surface to be hydrophilic.

### 2.3. Enzyme Functionalization

A 2% glutaraldehyde and a 10 U μL^−1^ GOD (from Toyobo Co., Ltd.) were mixed in a volume ratio of 1:1. Then, 3 μL of the mixture was placed evenly on the surface of the front end of the working electrode, with an area of 2 mm in width and 10 mm in length. The sensor was then dried in a refrigerator at 4 °C overnight. On the second day, it was taken out of the refrigerator and incubated with buffer at room temperature for about 1 h before sensing measurement.

### 2.4. Sensing Measurements

The measurement of the tongue depressor sensor for H_2_O_2_ or glucose was performed using a potentiostat CHI660e at room temperature. A constant potential of 0.6 V was applied between the working versus the reference electrode. First, 90 μL of phosphate buffer (50 mM, pH 7.0) was placed on the sensor, and 10 μL of H_2_O_2_ or glucose was injected into the buffer droplet. A series of different concentrations were injected sequentially. The current-versus-time curves were recorded, and a calibration curve between the steady-state current response and the concentration of H_2_O_2_ or glucose was plotted. The cyclic voltammetry of the sensor was studied in a buffer solution containing 2 mM of H_2_O_2_ or glucose, or without H_2_O_2_ or glucose. A small volume of glucose in water with a high concentration was added into the artificial saliva to mimic the saliva environment of diabetic patients. It was noticed that the slight dilution of the saliva with water had almost no change on the current baseline, compared with that of saliva.The rise in current was then recorded. For the comparision of the sensor with a spectrophotometric method, saliva was added to the solution of glucose catalytic enzyme, catalase, and 4-aminoantipyrine at room temperature for 15 min. After the reaction, a colored compound was formed, and the characteristic absorption peaks at 505 nm were detected by a spectrophotometer.

## 3. Results and Discussion

**Figure 1:**
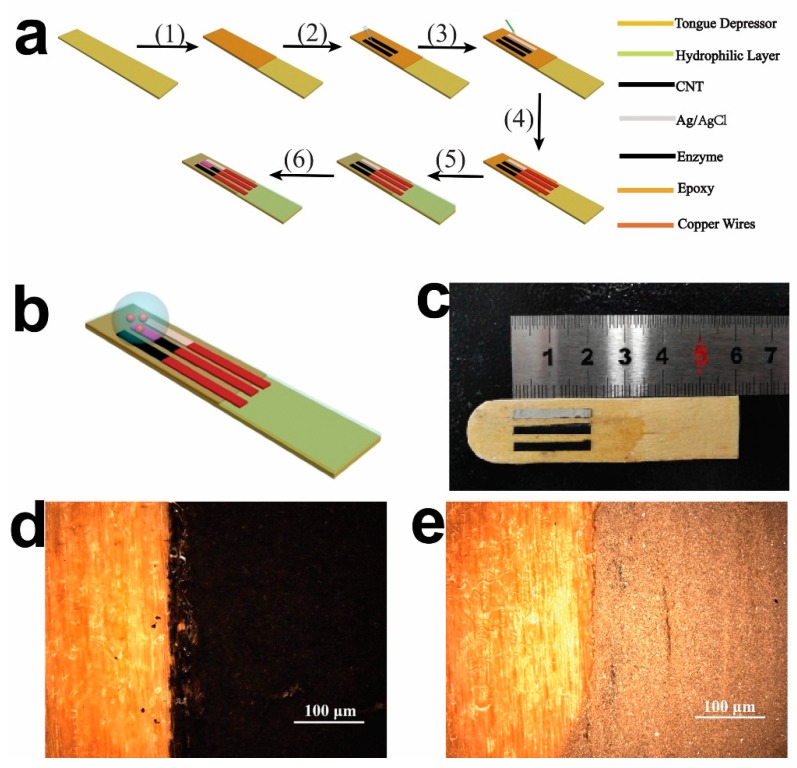
Images of the tongue depressor based amperometric sensor. (**a**) A schematic illustration of the preparion procedure for the sensor. Step (1): Painting epoxy; step (2): Printing carbon nanotubes (CNTs); step (3): Printing Ag/AgCl; step (4): Connecting to copper wires; step (5): Hydrophilic treatment; and step (6): Immobilizing enzymes. (**b**) A schematic illustration of the configuration of the sensor; (**c**) A camera image of the sensor; (**d**) An optical image of the carbon graphite electrode; (**e**) An optical image of the Ag/AgCl electrode.

Figure 1 shows a schematic illustration and images of the tongue depressor-based sensor. Figure 1a shows a schematic illustration for the sensor preparation. A tougue depressor was first painted with epoxy for insulation from water (1). Then, CNTs were printed on the substrate (2), followed by the printing of Ag/AgCl ink (3). Coppor wires were used to connect the electodes with the potentiostat (4). The sensor was then treated with UV ozone to change the surface property from being hydrophobic to be hydrophilic (5). After this, enzymes were immobilized on the working electrode (6). As can be seen in Figure 1b, all three electrodes were printed onto the tongue depressor in parallel and covered by a buffer droplet. The analytes were added into the buffer droplet and diffused to the electrode surfaces to induce the oxidation, reduction, and electron transfer on the electrodes. The camera image (Figure 1c) shows the working, reference, and counter electrodes successfully painted onto the surface of the tongue depressor in parallel. The electrodes are thin, straight, and have relatively sharp edges. The two electrodes in black color are the CNT electrodes that function as the working and counter electrodes. The grey color is the Ag/AgCl electrode that functions as the reference electrode. The optical images show the boundary between the wooden substrate and the CNT electrodes (Figure 1d), and the boundary between the wooden substrate and the Ag/AgCl electrode (Figure 1e). These images show that all the electrodes had uniform geometric shapes, and were successfully printed on the surface of the tongue depressor.

The detection mechanism is based on an enzymatic reaction. Glucose is oxidized by O_2_ to produce glucolactone H_2_O_2_ by GOD.

$$\text{Glucose}+{\text{O}}_{2}\overset{\text{GOD}}{\to }\text{Glucolactone}+{\text{H}}_{2}{\text{O}}_{2}$$

**Figure 2:**
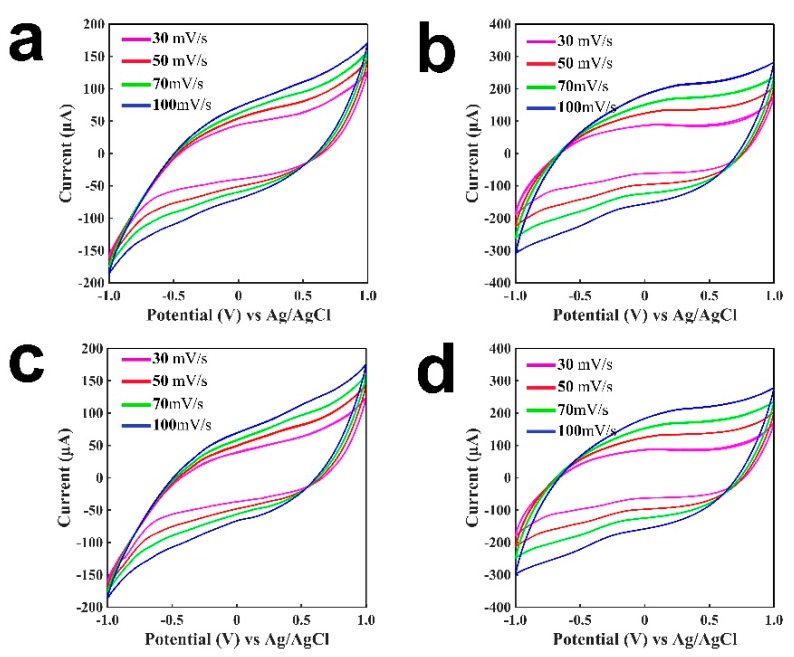
Cyclic voltammetry of the tongue-depressor amperometric sensors (from the second cycle). (**a**) A tongue depressor-based sensor without the immobilization of enzymes for buffer; (**b**) A tongue depressor-based sensor without the immobilization of enzymes for H_2_O_2_; (**c**) A glucose oxidase (GOD)-immobilized tongue depressor-based sensor for buffer; (**d**) A GOD-immobilized tongue depressor-based sensor for glucose.

H_2_O_2_ reacts at the working electrode at a positive potential: H_2_O_2_ − 2e^−^ = 2H^+^ + O_2_↑. From the amount of generated H_2_O_2_, the concentration of glucose can be determined. To explore the detection performance of H_2_O_2_ or glucose at different potentials, cyclic voltammetry was studied for the tongue depressor-based sensors, as shown in Figure 2. Figure 2a presents the cyclic voltammogram of the sensor for 2 mM H_2_O_2_ in the buffer. Figure 2b shows the cyclic voltammogram without H_2_O_2_ in the buffer. Figure 2c shows the cyclic voltammograms of GOD-immobilized sensor for 2 mM glucose in buffer. Figure 2d shows the cyclic voltammogram without glucose in the buffer. GOD catalyzed the oxidation of glucose to generate H_2_O_2_, which was then oxidized/reduced at the working electrode to generate a current change. From the comparison of Figure 2a,b, and from the comparision of Figure 2c,d, we can see that in the presence of H_2_O_2_ or glucose, the currents are higher than that without H_2_O_2_ or glucose at the same potential. The patterns for curves under 30 mV/s to 100 mV/s scanning rates were slightly different, as shown in both Figure 2a,c. This may be due to the different oxidation/reduction processes caused by different scanning rates, and the current is proportional to the squre root of the scanning rate. Comparing the current responses at a same potential in Figure 2a,c, it exhibits that glucose generated a smaller response than that of H_2_O_2_. This was expected, as H_2_O_2_ was oxidized/reduced directly on the electrode, whereas glucose went through the enzymatic reaction to generate H_2_O_2_, and H_2_O_2_ was oxidized/reduced on the electrode afterwards. The patterns of the curves are associated with the properties of the electronic inks, and these curves have similar patterns. As can be seen from the curves, with the increase of the potential, the current became larger. A potential of 0.6 V was chosen in this study, as this was sufficient for the oxidation or reduction process of H_2_O_2_.

**Figure 3:**
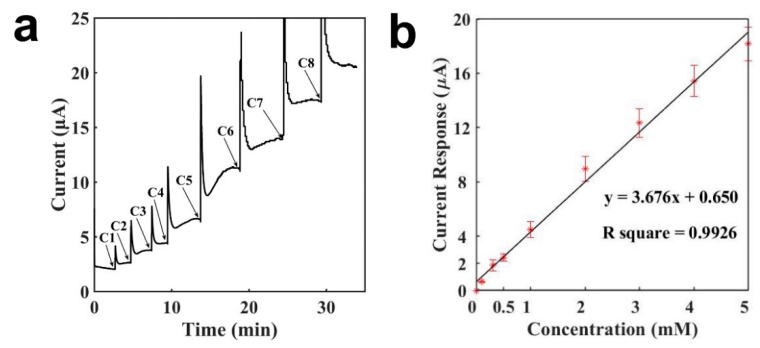
Characterization of the tongue-depressor amperometric sensors for H_2_O_2_ (phosphate buffer: 90 μL, droplet of H_2_O_2_ of different concentrations: 10 μL). (**a**) Current-versus-time curve for the detection of H_2_O_2_; (**b**) Calibration curve for the detection of H_2_O_2_. C1: 0.1 mM, C2: 0.2 mM, C3: 0.2 mM, C4: 0.5 mM, C5: 1 mM, C6: 1 mM, C7: 1 mM, C8: 1 mM.

Figure 3a presents the current-versus-time signal response curve of the tongue depressor sensor for detecting H_2_O_2_. When the current baseline with buffer was stable, H_2_O_2_ was injected into the buffer droplet. Then it diffused and reached the surface of the electrode, where it was then oxidized and generated a current response. When the entire diffusion and oxidation process became stable, the current arrived at a new steady state. The sensor response for H_2_O_2_ was rapid, and the entire detection took less than 5 min. At lower concentrations, the sensor response was even quicker, owing to the shorter diffusion process and reaction time. At C1 and C2, it took less than 1 min for the sensor to reach a stable current. The current response increased as the concentration of H_2_O_2_ increased, and it was proportional to the H_2_O_2_ concentration. As shown in Figure 3a, from C5 to C8, the current increases to the additions of 1 mM H_2_O_2_ were similar. Figure 3b illustrates the calibration curve for sensing H_2_O_2_. As can be seen in the figure, a linear relationship was obtained between the current response and the H_2_O_2_ concentration, with a concentration range of 0.1–5 mM, a slope of 3.676 μA mM^−1^, and an R^2^ of 0.9926. The detection limit of the tongue depressor biosensor for H_2_O_2_ was calculated to be 4.7 μM, 6.12 μM, and 4.08 μM (signal-to-noise ratio of 3), with an average of 4.97 μM and a standard deviation of 1.04 μM. The error bars were from repetitive experiments made by three different electrodes. It can be seen that the signal error between different electrodes is less than 15%. The results demonstrate that a tongue depressor-based sensor showed a rapid and sensitive detection of H_2_O_2_. The findings point to the possibility of constructing various biosensors based on tongue depressors via the immobilization of bioreceptors, such as enzymes, peptides, and antibodies.

**Figure 4:**
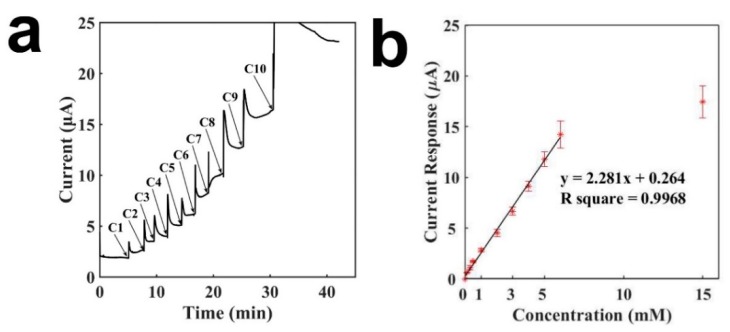
Characterization of the tongue-depressor amperometric sensors for glucose (phosphate buffer: 90 μL, droplet of glucose of different concentrations: 10 μL). (**a**) Current-versus-time curve for the detection of glucose; (**b**) Calibration curve for the detection of glucose. C1: 0.1 mM, C2: 0.2 mM, C3: 0.2 mM, C4: 0.5 mM, C5: 1 mM, C6: 1 mM, C7: 1 mM, C8: 1 mM, C9: 1mM, C10: 9 mM.

Figure 4 presents the characterization of the GOD-immobilized tongue depressor sensor for the detection of glucose. The immobilized GOD catalyzes the oxidation of glucose to generate H_2_O_2_. Therefore, through the detection of H_2_O_2_, glucose can be detected indirectly. Similarly, a buffer droplet was first placed on the sensor, and after the current baseline achieved a stable status, a series of glucose solutions were added sequentially to result in the increases of current. Figure 4a presents the current-versus-time curve for the detection of glucose. The sensor exhibited a rapid measuring time. Although it took around 5 min to detect 6 mM glucose, it could detect 0.3 mM glucose within 1 min. As the glucose concentration increased, the current response increased accordingly. As shown from C5 to C9 in Figure 4a, the biosensor showed similar current increases when adding several times of 1 mM glucose. Figure 4b presents the calibration curve of the tongue depressor biosensor for detecting glucose. A linear relationship was obtained between the current response and the glucose concentration, with a concentration range of 0.1–6 mM, a slope of 2.281 μA mM^−1^, and an R^2^ of 0.9968. The signal saturated at a concentration of 15 mM, whicg may be because of the saturation of the enzymatic reaction. The detection limit of the tongue depressor biosensor for glucose was calculated to be 7.3 μM, 10.96 μM, and 8.76 μM (signal-to-noise ratio of 3), with an average of 9.01 μM and a standard deviation of 1.84 μM. The results presented herein demonstrate that the tongue depressor-based biosensor has been successfully constructed and can detect glucose sensitively and rapidly. The sensing performances were comparable to the detection ranges and detection limits from other reports in the literature by using different electronic and substrate materials [30,31].

**Figure 5:**
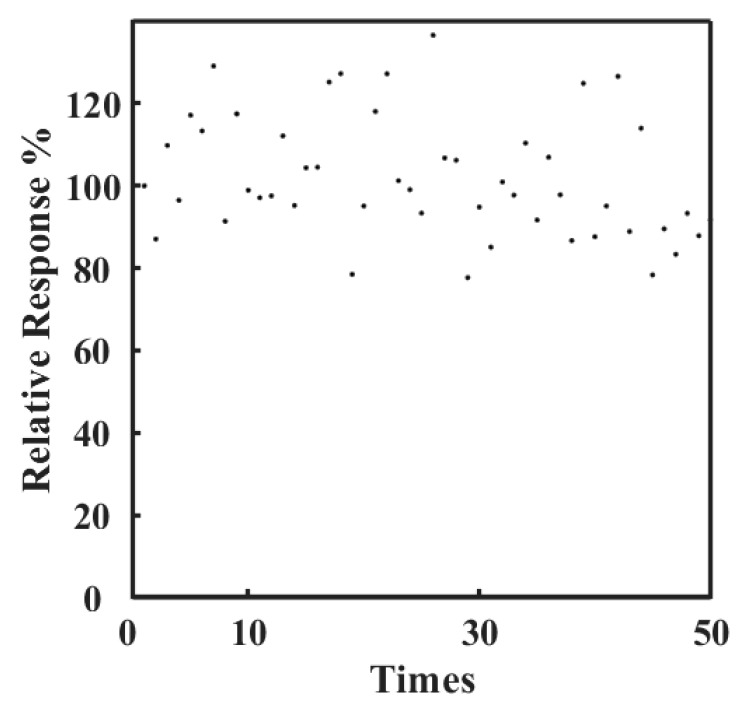
Stability of the tongue depressor amperometric sensors for glucose (phosphate buffer: 180 μL, droplet of glucose of 50 mM: 20 μL). Relative response (%): Normalizing the current reponse for the detection of glucose to the initial response.

Figure 5 shows the working stability of a tongue depressor glucose sensor. The sensor was studied for measuring a same glucose concentration for more than 50 times, where 20 μL of 50 mM glucose was added to 180 μL buffer and the current response was recorded. The response of the first detection is regarded as 100%, and the following results are showed in percentages based on the first response. It can be seen that the sensor maintained an excellent response for about 90% of the initial signal after detecting glucose for 50 times. This demonstrates that the sensor has an excellent working stability for the detection of glucose continuously.

**Figure 6:**
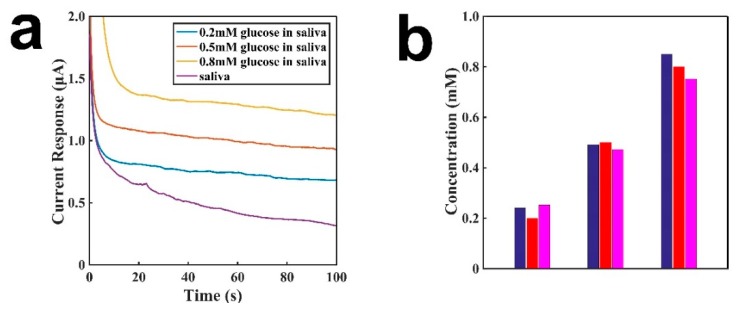
Comparison of the sensor and a spectrophotometer for the detection of different concentrations of glucose. (**a**) The baseline of saliva with different concentrations of glucose in saliva; (**b**) The comparison between the sensor and a spectrophotometer for the detection of different concentrations of glucose. Blue is from a spectrophotometer, red is the true concentration in saliva, and pink is from a sensor.

Figure 6 shows the comparison between tongue depressor amperometric sensors and a spectrophotometer. The actual concentrations were 0.2 mM, 0.5 mM, and 0.8 mM. Figure 6a show the graph of the sensor for detecting the concentration of glucose in saliva. Different concentrations of glucose in 200 μL of saliva were dropped onto the electrode surface. After the addition of glucose and waiting for 5 min to decompose glucose into H_2_O_2_, the concentration of H_2_O_2_ became uniform on the electrode in saliva, and the current baseline was detected for 100 s at 0.6 V. As shown in the figure, the signal is well correlated with the concentration of glucose. The glucose’s concentration in saliva was derived from the calibration curve in Figure 4. Figure 6b shows the comparision of the detection performances by using the sensor (pink) and a spectrophotometer (blue). The blue is the result from a spectrophotometer. As shown in the figure, both methods show comparable results, which are about the same as the standard concentrations of glucose. These results demonstrate that the sensor is an effective detection tool for measuring glucose concentrations in saliva.

**Figure 7:**
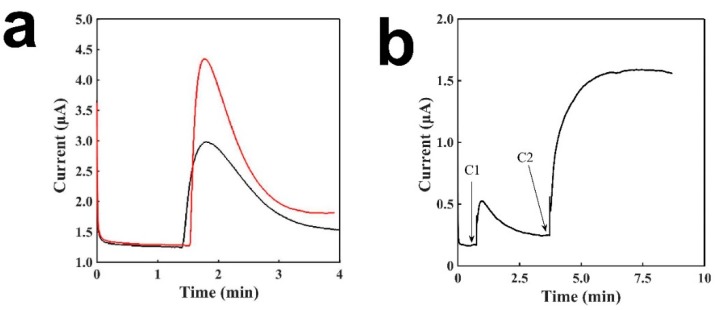
Characterization of the tongue depressor amperometric sensors for saliva. (**a**) The signal of the sensor with enzyme to H_2_O_2_ (red) and glucose (black). A total of 5 μL of 10 mM glucose solution was added to 195 μL of artificial saliva; (**b**) The signal responses to two different concentrations of glucose in saliva, C1: 0.048 mM and C2: 0.714 mM.

Figure 7 presents the current signal after the the addition of saliva containing glucose on the sensing electrode. According to previous research, the average glucose concentration of healthy people is 78.7 ± 9.2 μM for males and 80.4 ± 7.9 μM for females; while for diabetic patients, it is 201.9 ± 34.9 μM for males and 175 ± 22.3 μM for females [32]. We can see that the difference of glucose concentration between the healthy and diabetic groups is around 100 μM. We believe that this test to see current change against a concentration of about 50 μM of glucose in saliva should be able to prove our sensor’s capability in distinguishing between healthy and diabetic individuals. Saliva (195 μL) was placed onto the sensor to serve as the buffer solution; after the baseline had stabilized, 5 μL of H_2_O_2_ or 10 mM glucose was added to simulate the increase in glucose concentration for a diabetic patient, compared with that of a healthy individual. As shown in Figure 7a, we can see a clear rise of the current signal to glucose, and the signal of H_2_O_2_ is slightly larger than that of glucose, which may due to the catalytic conversion rate of glucose by the enzyme and the diffusion issue by the enzyme matrix. Figure 7b shows the the detection of different concentrations of glucose, and it can be seen that a higher concentration of glucose shows a much clear larger signal. The results proves that our sensor can distinguish different concentrations of glucose in saliva, and can distinguish between healthy people and diabetic patients easily.

## 4. Conclusions

In this work, we have shown the development of a tongue depressor-based biosensor for the first time, and its sensitive, rapid, and reliable determination of glucose in both phosphate buffer and real human saliva. The sensor construction method is simple, with CNTs dissolved in toluene, CNT and Ag/AgCl inks printed onto the surface of a tongue depressor, and GOD immobilized on the sensor. The detection process was easy-to-operate, cost-effective, and quick. The sensor showed a bro ad detection range and a rapid measuring time for glucose. The sensor performance was reliable. The low cost and portability of the substrate lend itself to be used in disposable devices, and make the proposed sensor extremely suitable for at-home tests or on-site medical examination. The noninvasive and disposable nature of the biosensor template improve the user experience, eliminate the chance of cross-infection, and also mean that it would be suited for use in examinations where there are concerns about the risk of infectious disease. Its practicability has also been proved in real human saliva test. This work may open up new avenues for developing various other tongue depressor-based biosensors dedicated to healthcare applications.
